# Histopathological and Immunohistochemical Features of Small to Big Satellite Nevus Uncover the Nevogenesis of Large/Giant Congenital Melanocytic Nevus

**DOI:** 10.1155/2022/9024548

**Published:** 2022-12-05

**Authors:** Jieyu Gu, Boxuan Wei, Bowen Gao, Ran Duan, Lingling Sheng, Danning Zheng, Yongyang Bao, Feng Xie

**Affiliations:** ^1^Department of Plastic and Reconstructive Surgery, Shanghai Ninth People's Hospital, Shanghai Jiao Tong University School of Medicine, Shanghai 200011, China; ^2^Department of Pathology, Shanghai Ninth People's Hospital, Shanghai Jiao Tong University School of Medicine, Shanghai 200011, China

## Abstract

The nevogenesis of large/giant congenital melanocytic nevus (lgCMN) is a complex biological process including several integral prenatal stages. Limited by ethical concerns, the debate of whether lgCMN develops from the epidermis to the dermis or in the opposite direction remains controversial. With the present study of the accompanying satellite nevi, we tend to support that lgCMN develops from epidermis to dermis. The satellite nevi were divided into 3 groups: big (diameter >10 mm), medium (>5 mm but ≤10 mm), and small (≤5 mm). Hematoxylin and eosin and immunohistochemical staining (SOX10, Ki67, and p16) were performed to compare the nevocyte infiltration depth as well as the positively stained rates among these satellite nevi. Compared to big satellite nevi, less deeply the nevocytes infiltrated the dermis, as well as more cells expressed SOX10 and Ki67 in the epidermis and fewer cells expressed p16 in the dermis of small satellite nevi. Additionally, two specimens were obtained from each of 4 patients who underwent serial resections of lgCMN at an average interval of 1.75 years to examine the histopathological changes. In the present study, satellite nevi of different sizes represent different stages of lgCMN from early to late, deepening our comprehension of the sequential stages of lgCMN nevogenesis. Initially, abnormal nevocytes seeded, proliferated, and spread along the epidermis. At rete ridges that protrude from the papillary dermis within the epidermis, some nevocytes formed nests and gradually penetrated into the dermis. Eventually, the nevocytes infiltrated the dermis and entered a homeostatic state. This study provides new evidence supporting the theory of epidermal-to-dermal nevogenesis in lgCMN.

## 1. Introduction

Large/giant congenital melanocytic nevi (lgCMN) are melanocytic lesions that cover the skin extensively at birth. Based on the predicted maximal adult size (PAS), lesions between 20 and 40 cm are classified as LCMNs, and lesions larger than 40 cm are classified as GCMNs [1]. How the nevocytes migrate and aggregate in the epidermis/dermis and eventually form melanocytic nevi during embryonic development remains unclear.

Currently, there are two hypotheses regarding the process of nevogenesis [2, 3]: (i) Unna's Abtropfung (“trickling down”) hypothesis, where mutated melanocytes in the epidermal basal layer proliferate abnormally and descend into the dermis, and (ii) Cramer's Hochsteigerung (“upward climbing”) hypothesis, where mutated precursors of melanocytes proliferate abnormally, occupy the dermis and finally form a nevus during migration towards the epidermis.

A study on nevogenesis demonstrated that acquired melanocytic nevus (AMN), which emerges postnatally, follows the “trickling down” hypothesis [4]. The similarities between CMN and AMN are as follows: (i) the histopathological architecture is characterized by gradual decreases in nevocyte size, pigmentation, and nest size deep within the dermis; however, the nevocytes infiltrate more deeply in CMN [5]; (ii) CMN and AMN may share the same global dermoscopic patterns and local features [6]. Thus, we proposed that the nevogenesis of CMN follows the same “trickling down” pattern as AMN. However, as a congenital disease, it is not feasible to observe the nevogenesis of lgCMN continuously in vivo limited by experimental ethics and techniques. In this study, the accompanying satellite nevi of lgCMN provide a new model for nevogenesis research.

The satellite nevi of lgCMN exist in various numbers and sizes, ranging from tens of centimeters to a few millimeters [1, 7]. The shared genetic mutation or fusion gene indicates that there might be homology between satellite nevi and the corresponding lgCMN [8, 9]. On the other hand, nevocytes were reported to transport throughout the circulation because benign nevocytes were discovered in blood vessels, lymphatics, and blood circulation in lgCMN [10–12]. Accordingly, the nevocytes in satellite nevi may originate from a single mutated nevocyte in lgCMN, transport to another skin site, and develop into a nevus. The process from a transported nevocyte to an aggregation of nevocytes in the epidermis/dermis of satellite nevi conforms to the nevogenesis of lgCMN during embryonic development. Via a histopathological investigation of satellite nevi of various sizes, our study reveals the nevogenesis from a single mutated nevocyte to the formation of a nevus.

We conducted hematoxylin and eosin (H&E) and immunohistochemical (IHC) staining to reveal the histopathological pattern of lgCMNs and satellite nevi. SOX10, a transcription factor found in neural-crest-derived cells, is indispensable for the specification, maturation, and maintenance of melanocytes [13]. Thus, it was used as a melanocytic lineage marker. Ki67, which is expressed only in cells with an active cell cycle, was used as a marker of proliferation [14]. p16 is a cyclin-dependent kinase inhibitor that negatively regulates the cell cycle and inhibits cell proliferation [15], and its expression is elevated in some melanocytic lesions [16–18].

Since big satellite nevi develop incrementally from small nevi, the nevogenesis of lgCMN can be inferred from satellite nevi of various sizes. This study was intended to uncover the histopathological characteristics of satellite nevi of various sizes, including the distribution of SOX10-positive cells, as well as the expression of the proliferation marker Ki67 and the cell cycle inhibitor p16, to reveal the nevogenesis from a single mutated nevocyte to lgCMN during embryonic development.

## 2. Materials and Methods

### 2.1. Selection of Patients and Specimens

Nineteen satellite nevus samples from 7 patients and thirty-nine lgCMN samples from 31 patients were included (see Table S1 in the Supplementary Materials). For 4 patients, two lgCMN samples were harvested at different times, with an average interval of 1.75 years. All satellite nevi were classified into 3 groups: big (diameter >10 mm), medium (>5 mm but ≤10 mm), and small (≤5 mm). All specimens were previously fixed in formalin and embedded in paraffin, and 4-*μ*m-thick sections were obtained for H&E and IHC staining. The study was approved by the Ethics Committee of Shanghai Ninth People's Hospital (Shanghai, China).

### 2.2. H&E Staining and Evaluation of the Nevocyte Infiltration Depth

Tissue sections were stained with H&E to examine the histopathological architecture, especially the nevocyte infiltration depth. To determine the infiltration depth in the dermis, H&E-stained sections of satellite nevi were observed under a microscope (Leica, Wetzlar, Germany) in a low-power field. Micrographs were taken using Image-Pro Plus 6.0 software (Media Cybernetics, Silver Spring, MD, USA). The nevocyte infiltration depth, defined as the maximum distance from the nevocytes at the dermal-epidermal junction (DEJ) to the deepest involvement in the dermis, was measured in microns. The results were derived from five randomly selected fields in each satellite nevi section.

### 2.3. IHC Staining

After deparaffinization and hydration, the sections were subjected to melanin bleaching (0.25% potassium permanganate for 1 minute and 0.5% oxalic acid for 1 minute) because excessive melanin would conceal the staining results. Heat-mediated antigen retrieval and blocking of endogenous peroxidase and nonspecific binding were performed according to the manufacturer's protocol. Then, the sections were incubated with primary antibodies against SOX10 (Abcam, ab227680, 1 : 100), Ki67 (Maxim, MX006, 1 : 200), and p16 (Maxim, MX007, 1 : 200) at 37°C for 1 hour. Then, the samples were incubated with an HRP-conjugated secondary antibody (Abcam, ab205718 for antirabbit, ab205719 for antimouse) at room temperature for 30 minutes and were then visualized using 3,3′-diaminobenzidine (DAB) as a chromogen. Some sections contained vast amounts of melanin; thus, the red chromogen 3-amino-9-ethylcarbazole (AEC) was used on these sections instead. Nuclei were counterstained with hematoxylin. The sections were observed under a light microscope and recorded digitally. For double immunofluorescence staining, FITC-conjugated goat antirabbit IgG (Abcam, ab6717) and Cy3-conjugated goat antimouse IgG (Abcam, ab97035) were used as secondary antibodies. These sections were imaged under a fluorescence microscope (Olympus, Tokyo, Japan).

### 2.4. Evaluation of IHC Staining

The SOX10, Ki67, and p16 immunostained sections were imaged and analyzed under microscopic examination at 400× magnification. The positively stained rate is the percentage of positive cells relative to the total number of cells in each field [19, 20] and is expressed by the following formula:

(1) $$\begin{array}{c} \text{Positively stained rate}=\frac{\text{Positively stained cells }}{\text{Total number of cells}}\times 100\%. \end{array}$$

For each case, the average rate of positive cells was calculated in 5 fields. As the cell density was different between the superficial and deep dermis, these areas were assessed separately.

### 2.5. Statistical Analysis

A nonparametric Kruskal–Wallis test (for >2 unpaired groups) was applied to compare the positively stained rate of SOX10 or Ki67 in the epidermis of big, medium, and small satellite nevi. The Mann–Whitney *U* test was used to analyze the difference between two groups (such as the positively stained rate of p16 in the dermis of lgCMN and satellite nevi). Differences were considered statistically significant if the *p* value was <0.05.

## 3. Results

### 3.1. Nevogenesis Progressed from the Epidermis to the Dermis

**Figure 1:**
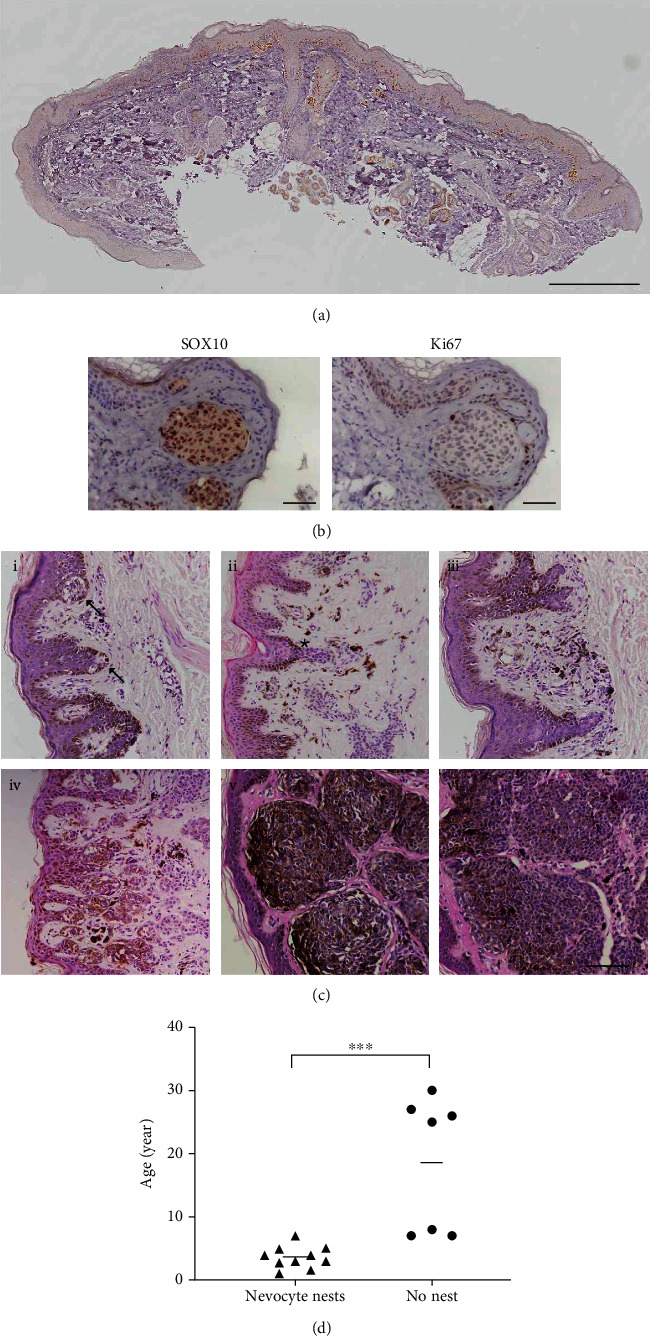
lgCMN develops from the epidermis to the dermis. (a) IHC staining of SOX10 in the smallest satellite nevus included in this study (scale bar = 100 *μ*m). (b) IHC staining of SOX10 and Ki67 in nevocyte nests in serial sections (scale bar = 50 *μ*m). (c) i. Nevocyte nests (indicated by the arrows) in the epidermal rete ridges and few nevocytes in the dermis of small satellite nevi; ii. the nevocyte nests expanded towards the dermis (indicated by the star); iii. nevocytes and melanin were presented in the dermis of satellite nevi but were limited to the superficial dermis; iv. a large number of nests penetrated the dermis; v. large nests in the dermis; vi. dispersed nevocytes and melanin in the dermis (H&E; scale bar = 100 *μ*m). (d) lgCMN patients without nests in the dermis appeared to be significantly older than those with nests in the dermis (^∗∗∗^*p* value =0.0002).

IHC staining of SOX10 in the smallest satellite nevus (2 mm in diameter) demonstrated that SOX10-positive cells were distributed in the epidermis but rarely deposited in the dermis, supporting the hypothesis that nevocytes first proliferate and spread in the epidermis (see Figure 1(a)). There were nests composed of aggregated nevocytes at the DEJ. SOX10 and Ki67 staining of serial sections verified the presence of Ki67-positive cells in these nevocyte nests, suggesting cell proliferation in the nests (see Figure 1(b)). The SOX10-positive cells in the dermis were associated with sweat glands, one of the histopathological features of CMN. The nests then penetrated the basal layer and infiltrated into the dermis while expanding. As a result, increased nevocytes and melanin were deposited in the dermis but were still limited to the superficial layer. Gradually, the nests dispersed into the deep dermis, resulting in a gradient distribution of nevocytes, with a high density in the superficial dermis and a low density in the deep dermis (see Figure 1(c)). In addition, the presence of nevocyte nests in the dermis was examined among patients of different ages. The result showed that lgCMN patients without nests in the dermis appeared to be significantly older (see Figure 1(d)).

### 3.2. Satellite Nevi of Various Sizes Represented the Dynamics of Nevogenesis

**Figure 2:**
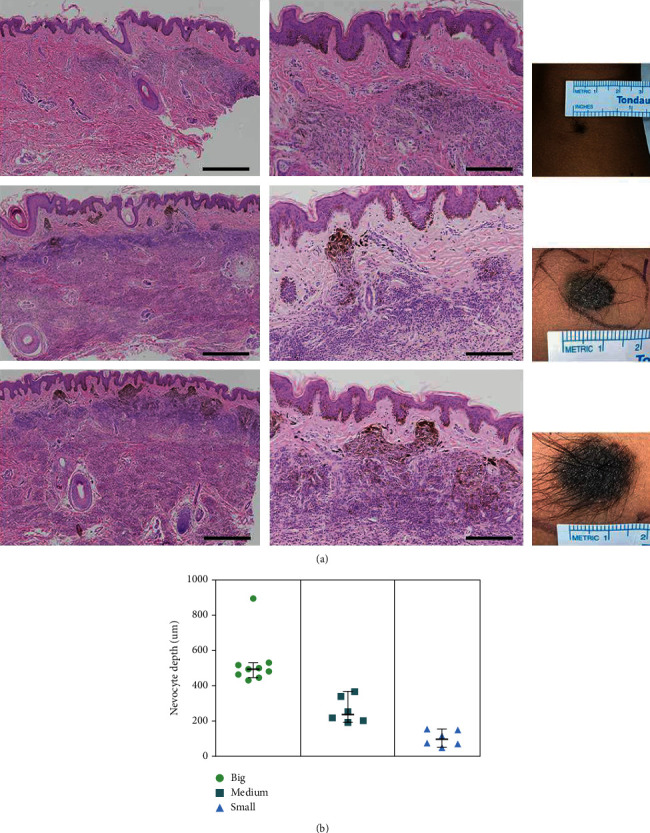
The infiltration depth of nevocytes varied with the size of satellite nevi. (a) H&E staining and clinical images of satellite nevi from small to big; the bigger the satellite nevus was, the deeper in the dermis where the nevocytes were deposited, with more melanin at the DEJ (scale bar = 500 *μ*m in the first column and 200 *μ*m in the second column; DEJ: dermal-epidermal junction). (b) The nevocyte infiltration depth of big, medium, and small satellite nevi.

**Figure 3:**
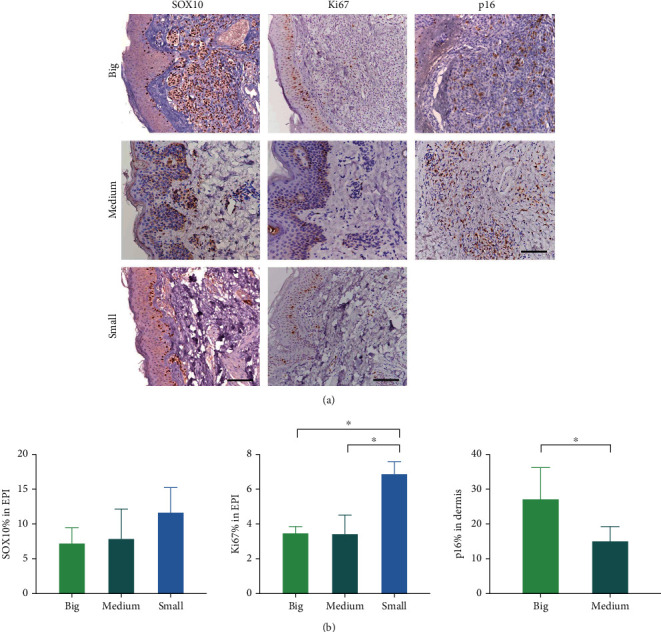
IHC staining of SOX10, Ki67, and p16 in big, medium, and small satellite nevi. (a) IHC staining of SOX10, Ki67, and p16 in big, medium, and small satellite nevi (scale bar = 100 *μ*m). (b) The positively stained rates of SOX10, Ki67, and p16 were compared among big, medium, and small satellite nevi (^∗^*p* value <0.05).

**Figure 4:**
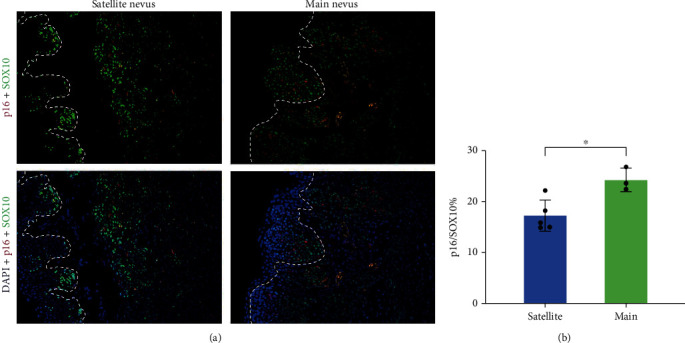
More nevocytes in lgCMN expressed p16 than in big satellite nevi. (a) Double immunofluorescence staining of SOX10 and p16 in lgCMNs and big satellite nevi (blue, DAPI; green, SOX10; red, p16; scale bar = 200 *μ*m). (b) Double-positive rates of SOX10 and p16 were compared between lgCMN and big satellite nevi (^∗^*p* value <0.05).

The infiltration depth of nevocytes varied with the size of satellite nevi (see Figure 2). In small satellite nevi, nevocytes were mainly located at the DEJ, with a low density. Medium satellite nevi exhibited a relatively higher density of nevocytes at the DEJ and dispersed nevocytes in the deep dermis. In addition to melanin at the basal layer of the epidermis, intradermal nevocytes also secrete melanin. In big satellite nevi, nevocytes not only penetrated more deeply but also had a higher density and more melanin. It can be concluded that the bigger the satellite nevus was, the more deeply the nevocytes infiltrated. IHC staining revealed that the average positively stained rate of SOX10 in the epidermis of small, medium, and big satellite nevi were 11.66%, 7.87%, and 7.23%, respectively (*p* value >0.05, see Figure 3, Table 1). The percentages of Ki67-positive cells in the epidermis were 6.85%, 3.43%, and 3.47%, respectively (*p* value <0.05, see Figure 3, Table 1). The smaller the satellite nevus was, the more Ki67-positive cells were in the epidermis. The percentage of p16-positive cells in the dermis was 26.98% in the big satellite nevi group and 14.80% in the medium satellite nevi group (*p* value <0.05, see Figure 3, Table 1). A comparison of the positively stained rate of p16 in the small group was excluded due to the rarity of nevocytes in the dermis. Furthermore, double immunofluorescence staining of SOX10 and p16 between big satellite nevi and lgCMN revealed that p16-positive nevocytes in the dermis of satellite nevi were significantly fewer than those in lgCMN (15.87% and 23.58% in big satellite nevi and lgCMN, respectively, see Figure 4).

### 3.3. lgCMN Eventually Reached a Homeostatic State

**Figure 5:**
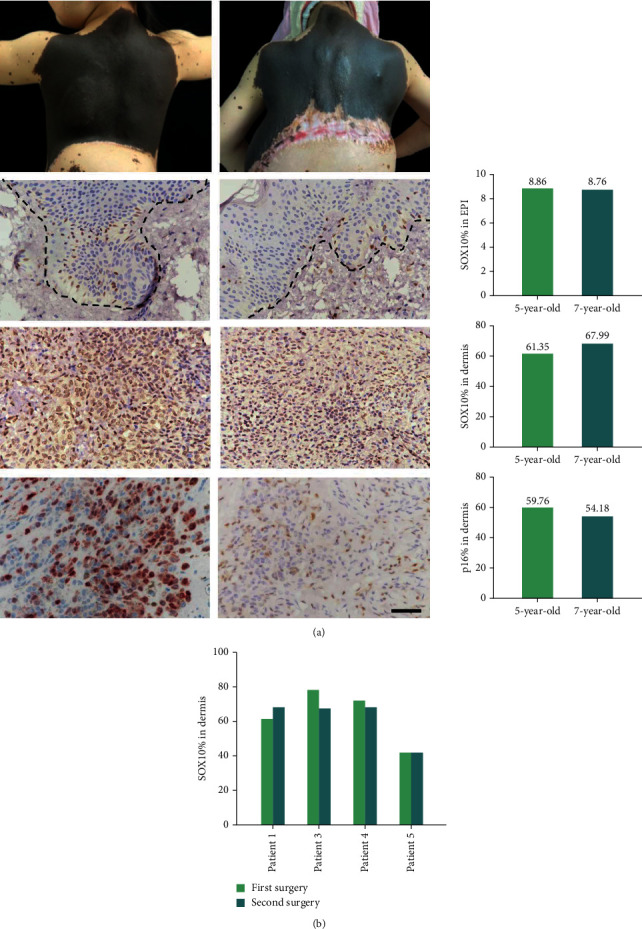
lgCMN eventually reached a homeostatic state. (a) Clinical images and IHC staining of SOX10 and p16 from one patient at the ages of 5 (first column) and 7 (second column) years. The positively stained rates of SOX10 in the epidermis and dermis and the positively stained rate of p16 in the dermis were compared (*p* value >0.05). (b) Comparison of the positively stained rates of SOX10 in the dermis obtained from two separate surgeries of four patients at a 2-year interval (*p* value >0.05).

Postnatally, the sizes of lgCMNs only increase with the growth and development of patients. The photos of patient 1 taken at the age of 5 and 7 showed no spreading of the nevus border. The IHC staining results were consistent with the clinical findings (see Figure 5(a)). The specimens harvested from two separate surgeries at a 2-year interval exhibited no significant change in the positively stained rates of SOX10 in either the epidermis or the dermis. In addition, there was no significant change in the positively stained rate of p16 in the dermis. The positively stained rates of SOX10 in the dermis were compared in the other three patients who had also undergone two surgeries, and no significant difference was found (see Figure 5(b)).

## 4. Discussion

The controversy over the hypotheses on nevogenesis focuses on whether nevocytes are first generated in the epidermis and then infiltrate into the dermis (Abtropfung pattern) or whether the lesions develop from dermis to epidermis during the migration and differentiation of precursors of melanocytes (Hochsteigerung pattern). The latter pattern corresponds with the widely accepted theory that melanocytes are derived from the neural crest and differentiate upon migration to the skin [21]. Besides, it was also reported a subepidermal noninvolvement zone at the DEJ in lgCMN and no abnormal melanocyte population overlying the epidermis [22, 23], which seems to support that the nevogenesis of lgCMN is independent of the epidermis. However, the limitation is that lgCMN with junctional involvement was excluded in those studies. In contrast, our study holds the opposite standpoint that the nevogenesis of lgCMN commences in the epidermis, in which the junctional lgCMN was included and regarded as a relatively early stage of nevogenesis, while the nevi with subepidermal noninvolvement zone were considered as a late stage. Additionally, Maguire et al. [24] also reported that nevocyte nests were mainly deposited in the epidermis with a few nevocytes extending from the papillary dermis to the deeper layer in a 5-month-old lgCMN patient, supporting that CMN originates in the epidermis at the beginning.

To verify, our study confirmed a histopathological similarity between satellite nevi and lgCMN (see Figure S1 in the Supplementary Materials). Thus, it was inferred that the nevogenesis of satellite nevi conformed with that of lgCMN in embryos, except that satellite nevi developed later than lgCMN, so they were smaller than lgCMN. Combined with previous findings of nevocytes in the blood or lymphatic vessels and the systemic circulation [2–5], our findings indicate that nevocytes in lgCMN could migrate through the systemic circulation to a distant skin location, where they would proliferate and eventually form satellite nevi during embryonic development. Therefore, this study reveals the process of lgCMN development by examining the histopathological features of small, medium, and big satellite nevi.

The smallest satellite nevus (2 mm in diameter) in this study exhibited distributed nevocytes along the epidermis and few nevocytes in the dermis (see Figure 1(a)), which is consistent with a previous study where abnormal melanocytes were focally clustered at the rete ridges of the epidermis in an 18-week-old aborted embryo [25]. Accordingly, our study indicates that abnormal nevocytes first colonize and spread along the basal layer during the early stage of nevogenesis, which supports the hypothesis that nevogenesis begins from the epidermis. Then, some nevocytes proliferate and form nests composed of aggregated nevocytes. IHC staining showed that almost all cells in the nests were positive for SOX10, and a few of them were positive for Ki67, indicating that nevocytes in the nests could proliferate (see Figure 1(b)). As the nest grows, it gradually penetrates the dermis until the entire nest descends and disperses into the dermis (see Figure 1(c)). The capability of nevocytes to migrate deeply into the dermis may be related to their escape from the control of keratinocytes [26, 27], which is the second step in nevogenesis. However, how nevocytes acquire this ability requires further study.

Based on their diameter, satellite nevi were classified as big (> 10 mm), medium (>5 mm but ≤10 mm), and small (≤5 mm). The infiltration depth of nevocytes varied with the sizes of satellite nevi. The larger the satellite nevus was, the more deeply nevocytes infiltrated, supporting the dynamics of nevogenesis from superficial to deep. IHC staining of SOX10 and Ki67 demonstrated that the smaller the satellite nevus was, the more Ki67-positive cells were in the epidermis, indicating that small satellite nevi were in the proliferative phase, which might correspond to the early stage of lgCMN development. p16 staining in the medium and big satellite nevi groups indicated that the bigger the satellite nevus was, the more cells expressed p16, indicating a state of cell cycle inhibition, which supports the hypothesis that satellite nevi of various sizes represent the dynamics of nevogenesis from early to late. Double immunofluorescence staining of p16 and SOX10 in big satellite nevi and lgCMN revealed more cell cycle-inhibited nevocytes in lgCMN than in satellite nevi (see Figure 4). The expression of p16 may contribute to the homeostatic state of lgCMNs, where the lesions no longer evidently grow. However, the underlying mechanism needs further study. To further prove the homeostatic state of lgCMN, we examined the positively stained rates of SOX10 and p16 between two separate specimens from the same patient at an interval of 2 years, which did not present a significant change. Combined with the above results, it was concluded that lgCMN eventually reached a homeostatic state, where the population and the component ratio of nevocytes remained stable.

**Figure 6:**
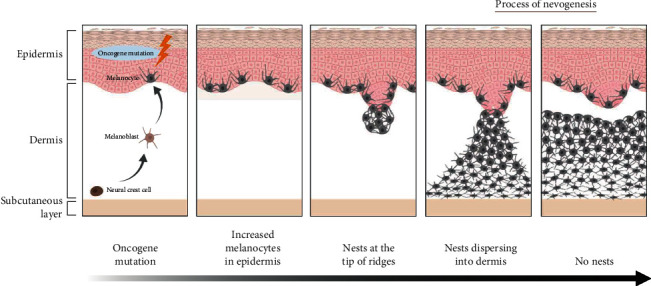
Schematic illustration of nevogenesis (created with BioRender). The mutated melanocyte first colonizes and proliferates along the basal layer of the epidermis, after which some of the nevocytes proliferate and form nests composed of aggregated nevocytes. As the nest grows, it gradually penetrates and disperses the dermis, forming a high density of round-shaped nevocytes in the superficial dermis and a low density of spindle-shaped nevocytes in the deep dermis.

From this histopathological study of lgCMN and satellite nevi of various sizes, we support the hypothesis that nevogenesis begins in the epidermis (see Figure 6). First, nevocytes abnormally proliferate and spread along the basal layer. At features such as rete ridges in the epidermis, some nevocytes aggregate, forming nests, which gradually expand and disperse into the dermis. During development, the positively stained rate of Ki67 decreases gradually, which means that their proliferation ability gradually decreases. Meanwhile, the expression of p16 in nevocytes in the dermis steadily increases, indicating a restraint on cell proliferation, which results in a balance in the number of nevocytes. Eventually, the nevus reaches a homeostatic state. This study provides new evidence for the theory of epidermal-to-dermal nevogenesis in lgCMN.

## Figures and Tables

**Table 1:** Positively stained rates of SOX, Ki67, and p16 in the epidermis/dermis of lgCMN and satellite nevi in various sizes.

	lgCMN	Satellite nevus			*p* value
		Big	Medium	Small	
SOX10% in epidermis	5.14	8.75			—
		7.23	7.87	11.66	>0.05

Ki67% in epidermis	4.24	5.47			—
		3.47	3.43	6.85	<0.05

p16% in dermis	—	—			—
		26.98	14.80	—	<0.05

p16/SOX10% in dermis	24.31	17.26			<0.05
		—	—	—	

## Data Availability

All data generated or analyzed during this study are included in this published article (and its supplementary information files).

## Supplementary Materials

Supplementary 1 Figure S1: satellite nevi were similar to lgCMN in clinical features and histopathological architecture. (A) The satellite nevi were similar to lgCMN in color, rugosity, and hypertrichosis. (B) The satellite nevi were similar to lgCMN in histopathological architecture. The nevocytes in the superficial dermis (second line) were round and secreted large amounts of melanin. The nevocytes in the deep dermis (third line) gradually became spindle-shaped and contained little melanin (H&E; scale bar = 200 *μ*m in the first row, 50 *μ*m in the second and third lines).

Supplementary 2 Table S1: clinical information of patients and specimens.
