# Supplementary figures and images for: Histopathological and Immunohistochemical Features of Small to Big Satellite Nevus Uncover the Nevogenesis of Large/Giant Congenital Melanocytic Nevus
Source: J Immunol Res. 2022 Dec 5;2022:9024548. doi: 10.1155/2022/9024548 (PMC9745446; doi:10.1155/2022/9024548)

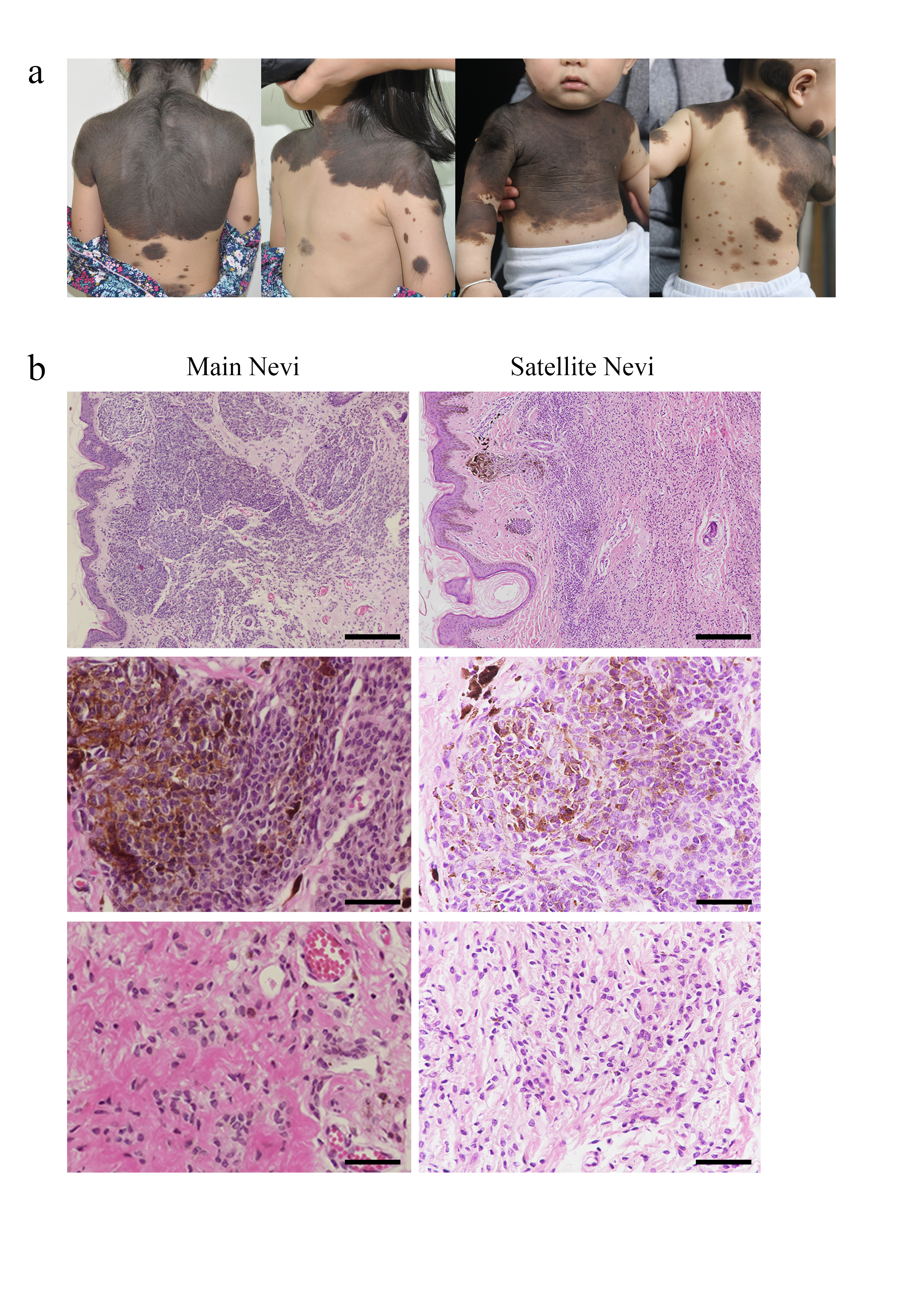

Supplement: Supplementary 1 — Figure S1: satellite nevi were similar to lgCMN in clinical features and histopathological architecture. (A) The satellite nevi were similar to lgCMN in color, rugosity, and hypertrichosis. (B) The satellite nevi were similar to lgCMN in histopathological architecture. The nevocytes in the superficial dermis (second line) were round and secreted large amounts of melanin. The nevocytes in the deep dermis (third line) gradually became spindle-shaped and contained little melanin (H&E; scale bar = 200 μm in the first row, 50 μm in the second and third lines). [file 9024548.f1.jpg]
