# Supplementary material for: Histopathological and Immunohistochemical Features of Small to Big Satellite Nevus Uncover the Nevogenesis of Large/Giant Congenital Melanocytic Nevus
Source: J Immunol Res. 2022 Dec 5;2022:9024548. doi: 10.1155/2022/9024548 (PMC9745446; doi:10.1155/2022/9024548)
Supplement: Supplementary 2 — Table S1: clinical information of patients and specimens. [file 9024548.f2.docx]

**Table S1. Clinical information of patients and specimens**

| **No.** | **Sex** | **lgCMN Location** | **Satellite Nevus** | **PAS** | **Age At Surgery (Year)** | **Sample Type** |
| --- | --- | --- | --- | --- | --- | --- |
| **1** | Female | Bolero | Yes | 40-60cm | 5 | lgCMN1 |
|  |  |  |  |  | 7 | lgCMN2 |
|  |  |  |  |  | 7 | Small Satellite1 |
|  |  |  |  |  | 7 | Small Satellite2 |
|  |  |  |  |  | 7 | Small Satellite3 |
| **2** | Female | Back | Yes | 40-60cm | 4 | lgCMN |
|  |  |  |  |  | 4 | Medium Satellite1 |
|  |  |  |  |  | 4 | Medium Satellite2 |
|  |  |  |  |  | 4 | Medium Satellite3 |
|  |  |  |  |  | 4 | Big Satellite |
| **3** | Female | Body | Yes | 40-60cm | 21 Months | lgCMN1 |
|  |  |  |  |  | 33 Months | lgCMN2 |
| **4** | Female | Scalp | No | 20-30cm | 25 | lgCMN1 |
|  |  |  |  |  | 26 | lgCMN2 |
| **5** | Female | Extremity | No | 40-60cm | 34 Months | lgCMN1 |
|  |  |  |  |  | 46 Months | lgCMN2 |
| **6** | Male | Body | Yes | >60cm | 3 | lgCMN |
|  |  |  |  |  | 3 | Big Satellite1 |
|  |  |  |  |  | 3 | Big Satellite2 |
|  |  |  |  |  | 3 | Big Satellite3 |
| **7** | Male | Trunk | Yes | 40-60cm | 13 Months | lgCMN |
|  |  |  |  |  | 13 Months | Big Satellite |
| **8** | Female | Breast | No | 20-30cm | Lost | lgCMN |
| **9** | Female | Head | Yes | 20-30cm | 27 | lgCMN |
| **10** | Female | Head | No | 20-30cm | 7 | lgCMN |
| **11** | Female | Head | No | 20-30cm | 3 | lgCMN |
| **12** | Female | Bathing Trunk | Yes | 40-60cm | Lost | Big Satellite |
|  |  |  |  |  | Lost | Medium Satellite |
|  |  |  |  |  | Lost | Small Satellite |
| **13** | Female | Extremity | No | 20-30cm | 13 | lgCMN |
| **14** | Female | Back | No | 30-40cm | 4 | lgCMN |
| **15** | Female | Back | No | 30-40cm | 29 Months | lgCMN |
| **16** | Female | Head | No | 20-30cm | 4 | lgCMN |
| **17** | Female | Back | Yes | 30-40cm | 29 Months | lgCMN |
| **18** | Female | Extremity | Yes | 20-30cm | 7 | lgCMN |
| **19** | Female | Extremity | Yes | >60cm | 6 | lgCMN |
| **20** | Female | Bathing Trunk | No | 30-40cm | 13 Months | lgCMN |
| **21** | Female | Breast/Belly | No | 20-30cm | 30 | lgCMN |
| **22** | Female | Back | Lost | 20-30cm | 12 | lgCMN |
| **23** | Male | Back | No | 40-60cm | 5 | lgCMN |
| **24** | Male | Extremity | Yes | 20-30cm | 18 | lgCMN |
| **25** | Male | Head | No | 20-30cm | 7 | lgCMN |
| **26** | Male | Head | Yes | 20-30cm | 3 | lgCMN |
| **27** | Male | Bolero | Yes | 20-30cm | 20 | lgCMN |
| **28** | Male | Back | No | 20-30cm | 5 | Big Satellite |
|  |  |  |  |  | 5 | Medium Satellite |
|  |  |  |  |  | 5 | Small Satellite |
| **29** | Male | Bolero | No | 20-30cm | 3 | lgCMN |
| **30** | Male | Head | No | 20-30cm | 25 | lgCMN |
| **31** | Male | Body | Yes | >60cm | 7 | lgCMN |
|  |  |  |  |  | 7 | Medium Satellite1 |
|  |  |  |  |  | 7 | Medium Satellite2 |
| **32** | Male | Extremity | Yes | 40-60cm | 4 | lgCMN |
| **33** | Male | Body | Yes | 40-60cm | 4 | lgCMN |
| **34** | Male | Back | Yes | 30-40cm | 4 | lgCMN |
| **35** | Male | Extremity | No | 20-30cm | 13 | lgCMN |
| **36** | Male | Head | Lost | 20-30cm | Lost | lgCMN |
| **37** | Male | Head | Yes | 20-30cm | 8 | lgCMN |
